# Editorial: Linking environmental exposure to toxicants and chronic disease

**DOI:** 10.3389/ftox.2024.1430316

**Published:** 2024-06-07

**Authors:** David R. Wallace, Aleksandra Buha Đorđević

**Affiliations:** ^1^ Oklahoma State University Center for Health Sciences, Department of Pharmacology and Physiology, Tulsa, OK, United States; ^2^ Department of Toxicology, Faculty of Pharmacy, University of Belgrade, Belgrade, Serbia

**Keywords:** non-communicable diseases, endocrine-disrupting chemicals, environmental contaminants, polycyclic aromatic hydrocarbons, TCDD, congenital disabilities, *in utero* exposure

Worldwide mortality caused by non-communicable diseases (NCD) has been rising. NCDs result from the combined effects of the genome and exposome, representing the continuum from all exposures *in utero* (or before) throughout the person’s lifespan. Exposure to environmental toxicants can take many forms. Indoor and outdoor exposure to air, water, and soil toxicants can impact human health. Research on the impact of endocrine-disrupting chemicals (EDC), persistent organic pollutants (POP), and toxic metals has significantly contributed to our understanding of the relationship between these agents and many NCDs. Little research has focused on health outcomes regarding human reproductive and NCD concerns following exposure to environmental toxicants. This edition, “Linking Environmental Exposure to Toxicants and Chronic Disease,” has compiled seven papers from leaders in their fields describing the action of environmental pollutants on the development of NCDs later in life. The NCDs can be neurodegenerative (Parkinson’s and Alzheimer’s), respiratory (asthma), *in utero* (hormonal changes), molecular (miRNA changes), or oncological (reproductive cancers).

The article by Helley et al. highlights mitochondrial dysfunction as a common pathway in Parkinson’s disease, emphasizing its critical role in neuronal function and proposing potential therapeutic strategies. They postulate that genetic mutations and environmental factors influence mitochondrial dysfunction. Their work was expanded on to include Alzheimer’s disease, in addition to Parkinson’s disease, and that is due to the multitude of environmental toxicants that we are exposed to daily. The complexity of these exposures and the resulting outcomes highlights the critical need for further research in this area (Nabi and Tabassum). The key findings of the paper include the recognition of environmental factors as significant contributors to neurodegenerative disorders, the emphasis on the role of environmental neurotoxins in diseases like Alzheimer’s and Parkinson’s, the acknowledgment of inconsistent findings in estimating risk levels for these disorders, and the call for more analytical studies and the identification of biomarkers for assessing previous exposure to environmental contaminants.

Inhalation of environmental toxicants is a major route of exposure for many toxicants. Once inhaled, many of these agents elicit inflammation that can manifest as a myriad of respiratory problems, including asthma and emphysema. The work of (Valdez et al.) examines our current understanding how inflammation induced by IL-13 affects the toxicity of polycyclic aromatic hydrocarbons (PAHs) in a 3D respiratory model for asthma using human bronchial epithelial cells, showing decreased barrier integrity, increased mucus production, goblet cell hyperplasia, and altered transcriptional biomarkers after exposure to BAP, with the IL-13 phenotype potentially having increased uncontrolled proliferation and decreased immune response. The IL-13 phenotype induced in HBEC cells significantly decreased barrier integrity and altered transcriptional biomarkers in response to BAP exposure. - The IL-13 phenotype showed increased potential for uncontrolled proliferation and decreased immune response after exposure to BAP compared to normal phenotype HBEC. - The study highlights the importance of considering pre-existing conditions like asthma in assessing susceptibility to chemical insults like PAHs.

In addition to direct exposure leading to respiratory inflammation, Golding et al. demonstrate the associations between grandparental exposures and asthma in grandchildren, with notable differences based on sex and specific grandparents involved, while highlighting strengths in data Research Topic and analysis. Stress from the death of a grandparent’s parent during childhood was associated with an increased risk of asthma in the grandchildren, especially if the paternal grandmother was involved. Smoking by grandparents during adolescence and tobacco smoking by the paternal grandmother during pregnancy was linked to a higher likelihood of asthma in the grandchildren. Combining all exposure variables increased goodness of fit, indicating potential synergistic effects between the exposures. *In utero*, exposure to the toxicants associated with smoke, such as wildfire smoke, suggests that the timing of exposure and the placental-adrenal-brain axis are critical in causing birth abnormalities (Lasley). Exposure to wildfire smoke during early gestation can lead to congenital disabilities, affecting adrenal development/function and neonatal temperament. Reduced cortisol levels during early gestation suggest a dampening of fetal adrenal steroid production due to smoke exposure.

The impact of environmental exposure to different toxicants has led to mixed molecular results and most likely depends on the toxicant. Exposure to 2,3,7,8-Tetrachlorodibenzo-p-dioxin (TCDD) impacts miRNA expression in the reproductive organs of adult female and male mice, linking these alterations to reproductive toxicity and potential transgenerational effects (Hall et al.). Exposure to TCDD altered the gestational period, impacted pup survival, and led to significant changes in miRNA expression in the reproductive organs of both male and female mice. Conversely, exposure to talc has received much attention lately as a potential carcinogen. The paper by Lynch et al. found suggestive evidence of no association between talc use and ovarian and endometrial cancers and insufficient evidence for cervical cancer, emphasizing the importance of considering biological plausibility and study quality. They report no associative evidence between perineal talcum powder application and ovarian, cervical, or endometrial cancer at human-relevant exposure levels. They conclude that insufficient evidence exists to establish a causal association between talc application and cancer confidently.

The comprehensive coverage of various Research Topic in this edition underscores the need for further research on exposure to environmental toxicants and the development of chronic diseases. The findings not only reveal direct toxicant-disease relationships, but also highlight a potentially alarming aspect-the ability of some toxicants to modify the genetic/molecular profile of individuals. This could lead to changes that can be inherited by future generations, raising intriguing questions about the long-term effects of environmental toxicant exposure and the need for further investigation.

